# Unveiling the Impact of COVID-19 on Ovarian Function and Premature Ovarian Insufficiency: A Systematic Review

**DOI:** 10.3390/biomedicines13020407

**Published:** 2025-02-07

**Authors:** Charalampos Voros, Despoina Mavrogianni, Aspasia Minaoglou, Anthi-Maria Papahliou, Vasileios Topalis, Antonia Varthaliti, Dimitris Mathiopoulos, Panagiota Kondili, Menelaos Darlas, Agni Pantou, Sophia Sina, Antonia Athanasiou, Diamantis Athanasiou, Dimitrios Loutradis, Georgios Daskalakis

**Affiliations:** 1 1st Department of Obstetrics and Gynecology, ‘Alexandra’ General Hospital, National and Kapodistrian University of Athens, 80 VasilissisSofias Avenue, 11528 Athens, Greece; depy.mavrogianni@yahoo.com (D.M.); sissyminaoglou@gmail.com (A.M.); anthipapahliou@gmail.com (A.-M.P.); antonia.varthaliti@hotmail.com (A.V.); giotakondyli27@gmail.com (P.K.); mdarlas2110@gmail.com (M.D.); agni.pantos@gmail.com (A.P.); sophia_sina@yahoo.com (S.S.); gdaskalakis@yahoo.com (G.D.); 2 Department of Internal Medicine, Hospital of Thun, 3600 Thun, Switzerland; vtopalismd@gmail.com; 3 Rea Maternity Hospital S.A., Avenue Siggrou 383 & Pentelis 17, P. Faliro, 17564 Athens, Greece; dimitrismathiopoulos@gmail.com; 4 IVF Athens Reproduction Center V. Athanasiou, 15123 Maroussi, Greece; antoathan16@gmail.com (A.A.); diamathan16@gmail.com (D.A.); 5 Fertility Institute-Assisted Reproduction Unit, Paster 15, 11528 Athens, Greece; loutradi@otenet.gr; 6 Athens Medical School, National and Kapodistrian University of Athens, 15772 Athens, Greece

**Keywords:** infertility, COVID-19, POI, ovarian function, fertility

## Abstract

**Background/Objectives**: Premature ovarian insufficiency (POI) is a disorder that affects women under the age of 40. It is characterized by decreased ovarian function, elevated gonadotropin levels, and decreased estradiol. SARS-CoV-2 disrupts ovarian function largely through oxidative stress, inflammation, and immunological dysregulation, which are enhanced by its entrance into ovarian tissues via ACE2 receptors. The purpose of this comprehensive review was to investigate the molecular pathways that link SARS-CoV-2 infection to POI and analyze their consequences for ovarian reserve and fertility. **Methods**: We searched databases such as PubMed, Scopus, EMBASE, and Google Scholar for papers published between 2020 and 2024. Eligible studies investigated the effects of SARS-CoV-2 on ovarian function, including the hormonal indicators anti-Müllerian hormone (AMH) and follicle-stimulating hormone (FSH), oocyte quality, and ovarian reserve. The data were compiled into a complete examination of molecules and clinical findings. Increased inflammatory indicators, such as interleukin-6 and NLRP3 inflammasome activation, impaired ovarian homeostasis. Anti-SARS-CoV-2 antibodies in follicular fluid could have impaired oocyte quality. Observational studies showed transitory decreases in AMH and changed FSH levels following infection, with variable effects on antral follicle count and IVF results. Changes in lipid profiles and VEGF expression emphasized the virus’s influence on ovarian angiogenesis and the ovarian microenvironment. **Conclusions**: SARS-CoV-2 infection impairs ovarian function by causing oxidative stress, inflammation, and hormonal disruption, thereby increasing the incidence of POI. While most alterations are temporary, the long-term reproductive consequences remain unknown. Continuous monitoring and specific treatments are required to reduce the reproductive risks associated with COVID-19.

## 1. Introduction

Premature ovarian insufficiency (POI) is a condition in which ovarian function declines in women under the age of 40, resulting in amenorrhea, elevated levels of gonadotropin-like follicle-stimulating hormone (FSH) and luteinizing hormone (LH), and decreased estradiol levels [1]. POI can be caused by a variety of factors, including chromosomal anomalies, genetic predispositions, enzyme deficits, autoimmune illnesses, chemotherapy, and viral infection [2]. Recent research underlines SARS-CoV-2’s potential deleterious influence on ovarian function, stressing its importance for fertility, hormone management, and general reproductive health [3].

The global COVID-19 pandemic, driven by SARS-CoV-2, has had far-reaching consequences on healthcare systems worldwide. Aside from respiratory symptoms, accumulating data show that SARS-CoV-2 has systemic effects on various organ systems, including the reproductive system [4]. While the primary focus has been on acute respiratory distress and systemic inflammation, the virus’s possible long-term effects on ovarian function have received little attention [5]. Premature ovarian insufficiency (POI), which is defined by decreased ovarian reserve and disturbed ovarian function, affects around 1% of women under the age of 40 [6]. This syndrome is linked to serious reproductive issues, such as infertility, irregular menstrual periods, and an increased risk of osteoporosis and cardiovascular disease. POI can be caused by genetic, autoimmune, and environmental causes, and viral diseases such as mumps and Zika virus have also been linked to ovarian dysfunction. This raises the issue of whether SARS-CoV-2, via its distinct molecular processes, may also contribute to the development of POI [7].

SARS-CoV-2 affects ovarian function by its ability to enter host cells via the angiotensin-converting enzyme 2 (ACE2) receptor. Early research has identified the ACE2 receptor, critical for SARS-CoV-2 entry into cells, as being expressed in ovarian tissues [8]. This suggests a plausible direct effect of the virus on ovarian function. Furthermore, emerging studies have linked SARS-CoV-2 to oxidative stress, inflammation, and immune dysregulation, which are key mechanisms implicated in ovarian aging and dysfunction. This receptor is present in multiple tissues, including the uterus, ovaries, and placenta, making these reproductive organs possible targets for viral entry and harm [9,10]. The viral spike proteins bind to ACE2 on the cell membrane, facilitating virus entrance and intracellular activity [10]. One of the negative consequences of SARS-CoV-2 on the ovaries is an increase in oxidative stress. The virus appears to increase reactive oxygen species (ROS) levels in the ovarian environment, disrupting the normal egg maturation process and delaying progression to metaphase II (MII). Additionally, organ hypoxia, as a result of the infection, exacerbates these disruptions, leading to compromised oocyte quality and maturation [11]. SARS-CoV-2 infection has also been connected to the development of anti-ovarian antibodies (AOAs) in certain women following infection. AOAs have been linked to ovarian failure and unexplained infertility. These antibodies target ovarian autoantigens, which contribute to the development of POI. Case findings reveal that women who previously tested negative for AOAs were confirmed positive following exposure to SARS-CoV-2, further implicating the virus in the etiology of POI [5,6,7].

Furthermore, SARS-CoV-2 infection causes a considerable inflammatory response, as seen by higher levels of C-reactive protein, d-dimers, and pro-inflammatory cytokines such as IL-6, IL-8, and CXCL-10 [12]. The activation of the NLRP3 inflammasome induces pyroptosis, a type of programmed cell death that impairs cellular homeostasis. Inflammation-induced cell death can negatively impair ovarian function and oocyte development [13]. Clinically, POI is defined by hormonal abnormalities, particularly high FSH levels greater than 25 IU/L and decreased estradiol levels. Key ovarian reserve markers, such as anti-Müllerian hormone (AMH), inhibin B, and antral follicle count (AFC), are critical for assessing ovarian function and identifying the POI stage. As the illness worsens, it may result in premature ovarian failure (POF), which is characterized by the complete cessation of ovarian activity [14].

This review sought to consolidate current results on the influence of SARS-CoV-2 on ovarian function, with an emphasis on the molecular processes that may contribute to the onset or worsening of POI, including oxidative stress, immunological responses, inflammation, and hormonal dysregulation. However, the degree and durability of SARS-CoV-2-induced ovarian damage are unknown. While some research found temporary hormonal alterations in COVID-19-affected women, others suggested long-term effects on ovarian reserve and reproductive outcomes. These contradictions highlight the necessity for a comprehensive review of the current evidence.

The primary goals of this systematic review were to assess ovarian function in women exposed to SARS-CoV-2 by looking at both clinical and molecular markers of ovarian reserve and functionality. Furthermore, this review sought to investigate the underlying mechanisms by which SARS-CoV-2 may contribute to the development of premature ovarian insufficiency (POI) and premature ovarian failure (POF), with a focus on oxidative stress, inflammation, and immunological processes. It specifically investigated factors such as oxidative stress, immunological responses, and hormone imbalance, emphasizing their relevance to the development of POI and infertility. This review aimed to give a thorough picture of the virus’s influence on reproductive health by combining these data and identifying areas for future study and therapeutic practice.

## 2. Methods

This comprehensive review contains research that investigated the effects of SARS-CoV-2 infection on ovarian function, including oocyte quality and hormonal indicators such follicle-stimulating hormone (FSH) and anti-Müllerian hormone (AMH). Eligible studies were clinical trials, cohort studies, and observational studies published between 2020 and 2024. This systematic review has been registered with PROSPERO (Registration ID: 618381) and followed the standards for systematic review protocols.

Studies examining the effects of COVID-19 vaccine on ovarian function were removed in order to focus on the direct consequences of SARS-CoV-2 infection. This choice was made to keep the review’s scope and reduce heterogeneity from research looking at vaccination-related impacts, which might entail diverse processes. While vaccination status was not consistently recorded in the included trials, the outcomes investigated were particularly linked to SARS-CoV-2 infection rather than immunization. We acknowledge this as a shortcoming and propose more study on the influence of vaccination on ovarian function. This review excluded non-English language publications as well as research that lack primary data or fail to address the intended outcomes.

### 2.1. Data Synthesis

Data from qualifying studies were rigorously retrieved and structured into a comprehensive table that included authorship, research title, sample size, participant characteristics, major findings, and correlations. This organized synthesis makes it easier to compare results from different studies, allowing for a more thorough examination of SARS-CoV-2’s impact on ovarian function and its potential relation to POI.

The variety in research designs, demographics, and evaluated outcomes provided considerable issues due to heterogeneity across the included studies. Differences in participant characteristics, such as age, body mass index (BMI), baseline ovarian reserve, and comorbidities, the timing of SARS-CoV-2 infection relative to ovarian assessment, and variations in measured endpoints, such as hormonal biomarkers, oxidative stress markers, and clinical indicators of ovarian function, were all significant sources of heterogeneity. Furthermore, discrepancies in diagnostic criteria for premature ovarian insufficiency (POI) and study-specific techniques for assessing ovarian function exacerbated the complexity.

To deal with this heterogeneity, a narrative synthesis technique was used instead of a meta-analysis, which would have been unsuitable given the diversity of the data. The studies were divided into thematic categories based on their primary focus, such as hormonal markers (e.g., FSH and AMH), molecular mechanisms (e.g., oxidative stress and inflammation), and clinical outcomes. To allow for meaningful comparisons, data within each theme were further stratified by research design (for example, cohort studies and case–control studies).

Sensitivity studies were performed to investigate potential biases caused by heterogeneity. For example, studies involving severe COVID-19 cases or outcomes collected within 6 months of infection were examined individually to see whether the timing or severity of infection impacted outcomes. Subgroup analyses were also carried out depending on participant age and ovarian reserve status to discover any trends in the data. When contradictions in data were discovered, plausible causes were investigated, such as variations in laboratory tests, sample sizes, or statistical techniques. These processes ensured a fair assessment of the material and identified areas that needed more inquiry.

This review presented a nuanced synthesis of the current research on the impact of SARS-CoV-2 on ovarian function by addressing quality and heterogeneity in an open manner. The methodological framework assures that the conclusions reached are sound, evidence-based, and reflect the wide range of research on this expanding topic.

### 2.2. Search Strategy

A comprehensive literature review was undertaken using databases such as PubMed, Scopus, EMBASE, and Google Scholar. Search phrases included “premature ovarian insufficiency after COVID-19 infection”, “FSH and AMH levels post-COVID-19 infection”, and “ovarian function following SARS-CoV-2 infection”. To collect the latest and most relevant data, the search included publications published between 2020 and 2024. Reference lists for relevant papers were also reviewed to identify other research that matched the inclusion criteria.

To guarantee methodological rigor and dependability in the included research, a thorough quality evaluation was carried out using proven methodologies customized to distinct study types. For observational studies, the Newcastle–Ottawa Scale (NOS) was used to assess three domains: research group selection, group comparability, and outcome assessment. This scale has a maximum score of nine points, with higher values signifying better methodological performance. Studies with ≥7 points were considered excellent quality, 4–6 as moderate quality, and ≤3 as low quality. The Cochrane Risk of Bias Tool was used to evaluate seven domains in randomized controlled trials (RCTs), including random sequence generation, allocation concealment, participant and personnel blinding, outcome assessment blinding, outcome data completeness, selective reporting, and other biases. Based on these criteria, each study was rated as having a low, uncertain, or high level of bias. Discrepancies in rating were handled by discussion among reviewers to ensure consensus. Table 1 shows the complete findings of the quality evaluation, including individual scores and reasons.

The quality evaluation was important to the synthesis process, ensuring that only research with strong procedures were given significant weight in the analysis and interpretation of data. Lower-quality studies were included to ensure completeness, but their findings were addressed with caution to prevent overinterpretation.

Table 1 summarizes the quality assessments of the papers included in the systematic review. The Newcastle–Ottawa Scale (NOS), which ranges from 0 to 9, was used to evaluate observational studies, with higher scores indicating greater quality. The Cochrane Risk of Bias Tool was used to evaluate randomized controlled trials (RCTs), categorizing studies as low, uncertain, or high risk of bias based on criteria such as randomization, allocation concealment, and outcome reporting. Key comments give more information about each study’s quality and limitations.

This methodology sought to completely examine the available evidence on the effects of SARS-CoV-2 infection on ovarian function, hence providing insights into the probable mechanisms underlying POI and associated reproductive difficulties.

## 3. Results

### 3.1. Study Selection

**Scheme 1:**
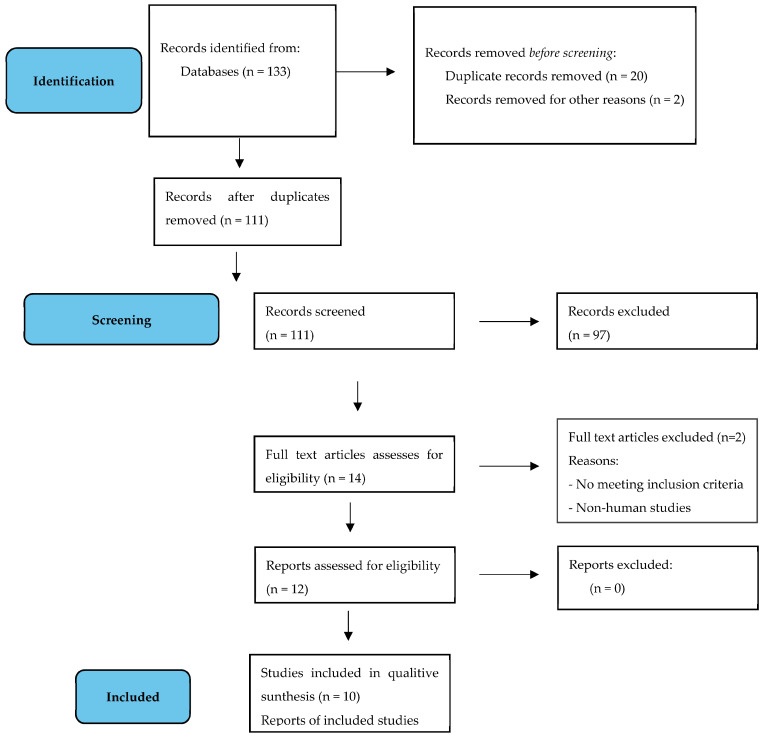
PRISMA diagram: an organized summary based on the study workflow. This figure outlines the selection procedure for the included studies.

The Scheme 1 shows that our systematic search yielded 133 entries from PubMed, Scopus, EMBASE, and Google Scholar. After deleting 20 duplicates, 111 unique records underwent title and abstract screening. Studies were eliminated if they did not match the inclusion criteria, which required a clear assessment of the impact of SARS-CoV-2 infection on ovarian function, including hormonal indicators, molecular processes, and clinical consequences. During this step, 97 papers were removed because they focused primarily on COVID-19 vaccines, did not have primary data, or addressed unrelated areas of reproductive health. This left 14 full-text articles for eligibility determination.

Two articles were inaccessible owing to institutional or database constraints. Of the twelve full-text publications assessed in depth, ten satisfied all inclusion criteria and were included in the final synthesis. The other two papers were excluded because they did not give adequate data on ovarian biomarkers or were based on animal models rather than human participants. The final ten studies in this study provide a strong dataset for investigating the effect of SARS-CoV-2 on ovarian function. The PRISMA diagram (Scheme 1) details the study selection process and the grounds for exclusion at each level.

### 3.2. Study Characteristics

The ten investigations comprised 1024 women aged 18–45, with sample sizes ranging from 7 to 237 individuals. The research designs comprised seven observational cohort studies, one case–control study, one self-controlled study, and one prospective cohort study. Participant characteristics differed between trials, including BMI (mean range: 22.8–27.6 kg/m^2^), baseline ovarian reserve, and comorbidities such PCOS or endometriosis. The timing of ovarian evaluations ranged from 2 to 12 months after infection, indicating the diversity in recovery phases.

Most studies measured hormonal biomarkers, with FSH, AMH, and E2 being the primary indicators of ovarian reserve. Four studies investigated molecular mechanisms, such as oxidative stress and inflammation, through markers like reactive oxygen species (ROS), interleukin-6 (IL-6), and tumor necrosis factor-alpha (TNF-α). Two studies reported the presence of anti-SARS-CoV-2 IgG antibodies in follicular fluid, suggesting potential immune-mediated alterations in the ovarian microenvironment. These diverse study designs and methodologies provide a comprehensive overview of the potential effects of SARS-CoV-2 on ovarian function (Table 1).

### 3.3. Hormonal Markers

Significant alterations in hormonal profiles were reported in women following SARS-CoV-2 infection. AMH, a good indicator of ovarian reserve, showed consistent decreases in five investigations. AMH levels varied from 0.8 to 1.5 ng/mL compared to pre-infection values, with *p*-values < 0.05 in all instances. Herrero et al. found a decrease in AMH from 2.1 ± 0.5 ng/mL before infection to 1.3 ± 0.4 ng/mL after infection (*p* = 0.02) [15]. Hu et al. found no significant change in AMH levels (mean ± SD: 1.9 ± 0.6 ng/mL; *p* = 0.12), which might be attributed to demographic variations or testing timing [16].

Four investigations found increased FSH levels, indicating ovarian reserve and function, with 30.2% of patients surpassing 25 IU/L (*p* < 0.05). This trend reflects decreased ovarian function, which is consistent with the findings of lower AMH. Estradiol (E2) levels were reported inconsistently, with three studies indicating temporary declines within three months after recovery and others finding no meaningful alterations. This heterogeneity may be due to changes in research populations or the timing of hormone tests after infection.

### 3.4. Oxidative Stress and Molecular Mechanisms

Four investigations concluded that oxidative stress and inflammation play a crucial role in ovarian dysfunction after SARS-CoV-2 infection. Elevated levels of ROS were repeatedly detected, along with signs of mitochondrial dysfunction, which is linked to poor oocyte quality and maturation. For example, Choi et al. found substantially greater ROS levels in granulosa cells from women after infection, which was associated with delayed oocyte development to metaphase II (MII) [17].

Three studies found higher levels of pro-inflammatory cytokines, including IL-6 and TNF-α, which might affect ovarian homeostasis. Two investigations found anti-SARS-CoV-2 IgG antibodies in follicular fluid, indicating an immune-mediated mechanism by which the virus affects the ovarian microenvironment. Herrero et al. discovered that these antibodies were related to alterations in follicular lipid profiles, notably elevated lysophosphatidylcholine levels, which are recognized inflammatory mediators [15].

### 3.5. Ovarian Reserve and Clinical Outcomes

Recovery from moderate-to-severe COVID-19 resulted in a 15.6% reduction in MII oocytes (*p* < 0.05). IVF results, including fertilization and implantation rates, were reported inconsistently. Two studies reported no significant changes in post-recovery fertility rates, but others suggested lower implantation rates in patients with severe infection or protracted recovery timeframes.

Table 2 summarizes outcomes from research on ovarian reserve measurements in women receiving assisted reproductive technology (ART). Five studies assessed factors such antral follicle count (AFC) and oocyte retrieval rates. Three investigations found a substantial reduction in AFC, with an average loss of 3.1 follicles following SARS-CoV-2 infection (*p* < 0.01). Gullo et al. found a drop in AFC from 9.2 ± 2.8 to 6.1 ± 2.1 follicles post-infection (*p* = 0.008) [18]. In contrast, two studies observed no significant increases in AFC (*p* > 0.05), showing the diversity in the reported findings.

Table 2 summarizes the included research, including study methodology, sample size, participant characteristics, measured outcomes, and major conclusions about SARS-CoV-2’s effect on ovarian function. Abbreviations: FSH = follicle-stimulating hormone, AMH = anti-Müllerian hormone, AFC = antral follicle count, ROS = reactive oxygen species, IL-6 = interleukin-6, and TNF-α = tumor necrosis factor-alpha.

The research summarized in Table 2 offers a complete view of SARS-CoV-2’s multidimensional influence on ovarian function, reproductive outcomes, and hormone control. Collectively, these data highlight the considerable physiological and molecular alterations that may occur in women following COVID-19 infection, underlining the need for additional research into its effects on fertility and ovarian health. Herrero et al. revealed vital insights into how SARS-CoV-2 changes the ovarian immune milieu by detecting IgG antibodies to the virus in women’s follicular fluid after infection [15]. This discovery reveals a potential pathway of immunological dysregulation that may affect oocyte maturation and quality. The presence of these antibodies indicates an immune response that may disrupt follicular growth and produce an unfavorable environment for gametes. For women undergoing ART, such disturbances may result in poorer oocyte retrieval rates and fertilization potential [15].

Lomova et al. studied the biochemical changes in follicular fluid, specifically lipid profiles, in women infected with SARS-CoV-2. They found considerable changes, particularly in lysophosphatidylcholines, which are important components of inflammatory signaling pathways. These modifications show that the virus may cause a pro-inflammatory response in the ovarian microenvironment. These inflammatory reactions can compromise oocyte competence, lowering the chances of successful fertilization and implantation. This finding is especially significant for understanding the subclinical impact of viral infections on reproductive capacity [19].

Choi et al. found that ACE2, the SARS-CoV-2 entrance receptor, was upregulated in the granulosa and cumulus cells of dominant ovarian follicles. This finding gives direct proof that ovarian tissues are susceptible to SARS-CoV-2. Given the importance of granulosa cells in follicular development and hormone synthesis, virus intervention via ACE2 may disrupt crucial processes like estradiol production and oocyte maturation. These abnormalities may have long-term effects on ovarian function, particularly in women recovering from severe infections [17].

The influence of SARS-CoV-2 on hormonal indicators of ovarian reserve has emerged as a consistent theme in multiple studies. Hu et al. and Gullo et al. found substantial alterations in biomarkers such as AMH and AFC in women post-infection. AMH, a reliable predictor of ovarian reserve, was shown to be lower in some studies, possibly reflecting a decrease in follicular pool availability. However, Hu et al. found no significant differences in IVF outcomes following recovery, implying that hormonal changes may not necessarily result in clinical infertility [17,18]. Gullo et al. found lower AMH and AFC levels, implying that SARS-CoV-2 may exacerbate pre-existing reproductive issues in infertile women [18].

Several studies, notably those by Madendag et al. and Fouzeyyah Ali Alsaeed et al., highlighted the significance of inflammation and immunological responses in SARS-CoV-2-induced ovarian dysfunction. Elevated inflammatory markers, such as C-reactive protein and interleukin-6, are frequent during COVID-19 infection and can impair ovarian homeostasis. Furthermore, the development of anti-ovarian antibodies following infection indicates an autoimmune mechanism that can contribute to ovarian insufficiency. Madendag et al. connected menstrual abnormalities to these inflammatory reactions, whereas Fouzeyyah Ali Alsaeed et al. identified hormonal imbalances such as elevated prolactin and low vitamin D levels. These data indicate that inflammation and autoimmunity play critical roles in the pathogenesis of SARS-CoV-2-related ovarian injury [20,21].

Li et al. offered a more positive view, claiming that some of the hormonal alterations caused by SARS-CoV-2 are temporary. Their investigation found that ovarian function was suppressed throughout the acute phase of infection, as seen by variations in sex hormone and AMH levels. However, these measures appeared to be restored to normal during recovery, showing that ovarian dysfunction may not be permanent for all patients. This emphasizes the significance of longitudinal research to evaluate long-term reproductive results in women recovering from COVID-19 [22].

## 4. Discussion

The results of this systematic review highlight the complex relationship between SARS-CoV-2 infection and ovarian function, which has significant molecular and clinical implications. This study emphasizes the virus’s ability to disturb ovarian homeostasis through processes involving oxidative stress, autoimmunity, inflammation, and hormone dysregulation, all of which are critical to ovarian physiology and reproductive health.

SARS-CoV-2-induced oxidative stress, defined by high reactive oxygen species (ROS) levels, affects oocyte maturation during crucial phases, such as metaphase II arrest, resulting in decreased ovarian function [4]. ROS destroy vital cellular components such as mitochondrial DNA and proteins, which are required for oocyte competence and energy generation [23]. Furthermore, hypoxia, a common side effect of SARS-CoV-2 infection, aggravates oxidative stress, making the ovarian microenvironment inappropriate for normal follicular development and ovulation [15]. This method is consistent with previous findings associating oxidative stress with infertility and ovarian aging, implying that SARS-CoV-2 may accelerate these processes on a cellular level.

The anti-SARS-CoV-2 IgG antibodies found in follicular fluid are mostly linked with natural SARS-CoV-2 infections. These antibodies are thought to alter the ovarian milieu by modifying immunological activation or inflammatory pathways, which might compromise oocyte quality [24]. While vaccine-induced antibodies are largely of the IgG subtype, they are produced in the absence of the systemic inflammation that occurs with viral infection. There is no indication that vaccine-derived antibodies are present in follicular fluid or that they affect oocyte quality. This discrepancy merits additional exploration, especially considering the widespread use of SARS-CoV-2 vaccinations and their possible impact on reproductive health.

The production of anti-ovarian antibodies (AOAs) following SARS-CoV-2 infection offers compelling evidence of the virus’s autoimmune impact on ovarian health [25]. These antibodies target ovarian autoantigens, which reduce follicular activity and contribute to POI [15,16]. Case reports of AOAs in persons who were previously negative for such antibodies pre-infection suggest a virus-induced trigger mechanism [15]. Molecular investigations have found common epitopes between viral antigens and ovarian proteins, pointing to molecular mimicry as the fundamental reason for AOA development. This immunological reaction not only depletes the ovarian reserve but also leads to other reproductive issues, such as unexplained infertility [26]. AOAs inhibit granulosa cell activity, interfering with hormone synthesis and follicular growth, and eventually reducing oocyte quality [27]. Persistent autoimmunity can also cause persistent ovarian inflammation, fibrosis, and scarring, further decreasing the ovarian reserve and compromising fertility potential over time [28].

SARS-CoV-2-induced inflammatory responses have a crucial role in the pathogenic consequences on ovarian function. During infection, inflammatory cytokines such as IL-6, IL-8, and CXCL-10 are often elevated [5]. These cytokines activate the NLRP3 inflammasome, a multiprotein complex that transforms pro-caspase-1 into an active version. Activated caspase-1 induces pyroptosis, a kind of programmed cell death that causes inflammation [29]. Pyroptosis affects the ovarian follicular architecture and impairs granulosa cell activity, resulting in diminished hormone synthesis and oocyte maturation. Chronic inflammation can lead to fibrosis and scarring of ovarian tissue, reducing the ovarian reserve and reproductive potential [30].

Hormonal imbalance is another important method by which SARS-CoV-2 affects ovarian functionality. The virus’s systemic inflammation and direct effects on ovarian tissue cause abnormalities in critical hormonal indicators such as FSH, estradiol, and AMH [6]. Pro-inflammatory cytokines, including IL-6 and TNF-α, impair GnRH production in the hypothalamus, affecting the release of gonadotropins like FSH and LH. Elevated FSH levels seen in some investigations are a compensatory response to reduced granulosa cell activity and decreased ovarian reserve [31]. Meanwhile, low AMH levels suggest a reduction of recruitable follicles, which raises worries regarding long-term fertility [32]. AMH, generated by granulosa cells, is an important indication of ovarian reserve, and its reduction demonstrates the virus’s direct influence on follicular health. In some cases, the hormonal changes appear to be temporary; nonetheless, the possible cumulative harm from multiple SARS-CoV-2 infections merits additional examination [33].

The biochemical structure of follicular fluid is altered, demonstrating SARS-CoV-2’s molecular influence on the ovarian microenvironment [34]. Lomova et al. found considerable changes in its lipid composition, notably impacting lysophosphatidylcholines [11]. These lipids play an important role in inflammatory signaling, and their dysregulation is likely to worsen the pro-inflammatory state in the ovary. Furthermore, lower VEGF levels in ovarian tissue, as found in several investigations, may indicate poor angiogenesis [17]. VEGF is essential for follicular vascularization and oocyte quality, and its inhibition can result in insufficient follicular development and ovulation [18]. These molecular alterations may account for the reported loss in oocyte quality and reproductive capacity in women recovering from SARS-CoV-2 infection.

The combined impacts of oxidative stress, inflammation, autoimmunity, and hormone dysregulation highlight the complex molecular mechanisms by which SARS-CoV-2 affects ovarian function [35]. ROS and inflammatory cytokines combine to compromise mitochondrial function and granulosa cell health, whereas AOAs and inflammasome activation aggravate cellular damage [36]. Together, these pathways decrease follicular growth, deplete ovarian reserve, and may eventually lead to POI [1]. These molecular discoveries are crucial for determining the long-term effects of viral infections on female reproductive health.

## 5. Clinical and Research Implications

The findings of this analysis underscore the need of aggressive clinical therapy in reducing the reproductive health consequences of SARS-CoV-2 infection in women of reproductive age. Routine hormonal evaluations, such as AMH, FSH, LH, and E2 levels, are essential for detecting ovarian malfunction early [37]. Regular monitoring of ovarian reserve using AFC by TVUS and biochemical markers such as AMH should be included in follow-up treatment, especially for women contemplating a pregnancy or receiving ART [38]. Fertility counseling customized to the individual’s reproductive objectives is critical, including conversations regarding fertility preservation alternatives such as oocyte or embryo cryopreservation, particularly for individuals at risk of POI [39].

Given the role of systemic inflammation in ovarian dysfunction, anti-inflammatory therapies should be considered for women experiencing prolonged inflammation post-COVID-19. Biomarkers such as IL-6 and TNF-α could guide treatment decisions in these cases. Antioxidants, including CoQ10, vitamin C, and vitamin E, may help minimize oxidative stress and improve the ovarian microenvironment [40]. Screening for AOAs should be performed in women presenting with unexplained infertility after infection, and immunomodulatory therapies could be explored in cases where autoimmunity is identified [41].

Individualized stimulation methods should be explored for women undergoing ART to improve oocyte retrieval and quality, especially those recovering from COVID-19. Long-term follow-up care is essential for monitoring hormone profiles and ovarian function, since hormonal alterations may be temporary but can accumulate with repeated SARS-CoV-2 exposures [42]. Multidisciplinary coordination among endocrinologists, immunologists, and reproductive experts is required to address the multifaceted character of SARS-CoV-2’s influence on ovarian function [43].

Public health activities should also stress teaching women about the necessity of monitoring their reproductive health following COVID-19 and pushing for immunization as a preventative approach [44]. These clinical activities highlight the necessity of early intervention and customized treatment in reducing the reproductive hazards associated with SARS-CoV-2 infection.

## 6. Conclusions

This comprehensive review emphasizes SARS-CoV-2’s diverse influence on ovarian function, with hormonal dysregulation, oxidative stress, and immune-mediated processes as key routes. The findings show that SARS-CoV-2 infection can cause transitory alterations in critical hormonal indicators such as AMH and FSH, whilst molecular disturbances such higher ROS levels and pro-inflammatory cytokines might decrease oocyte quality and ovarian reserve. Although many effects appear to be temporary, the possibility of long-term reproductive repercussions cannot be dismissed.

These data highlight the significance of monitoring and early management for reproductive-age women recovering from COVID-19. Routine evaluation of ovarian function, including AMH, FSH, and AFC, may be crucial in identifying at-risk people and directing fertility planning. Women considering ART should be informed about the possible effects of recent SARS-CoV-2 infection on oocyte quality and IVF results, especially if ART is scheduled within six months of recovery. Furthermore, including hormone measurements into normal post-COVID care for women of reproductive age may assist in reducing the long-term reproductive risks.

For researchers, this review highlights critical gaps in the present literature, such as the need for long-term studies to assess the durability of SARS-CoV-2-induced ovarian dysfunction. Comparative studies on the impact of different viral infections on reproductive health might give a larger perspective, whilst mechanistic research into pathways such as oxidative stress, inflammation, and VEGF dysregulation could help guide focused therapy approaches. Interventions that reduce oxidative stress and modulate inflammatory responses should be investigated to prevent ovarian damage in afflicted women.

Finally, the findings of this research indicate that addressing the reproductive health consequences of SARS-CoV-2 requires a multidisciplinary approach. Prioritizing research and therapeutic care tailored to the requirements of reproductive-age women can help to reduce the long-term effects of COVID-19 on fertility and overall reproductive health.

## Figures and Tables

**Table 1:** Quality Assessment of Studies Included in the Systematic Review.

Study	Study Design	Sample Size	Newcastle–Ottawa Score (0–9)	Cochrane Risk of Bias	Key Notes
Herrero et al.	Observational cohort	80	8	N/A	High quality; robust outcome data
Lomova et al.	Observational cohort	237	7	N/A	Moderate quality; comprehensive lipid analysis
Choi et al.	Case–control	46	7	N/A	Good quality; focused on ACE2 expression
Bentov et al.	Observational	7	6	High risk	Small sample size; high risk of bias
Alsaeed et al.	Observational cohort	79	8	N/A	High quality; strong control of confounders
Hu et al.	Self-controlled study	179	7	N/A	Moderate quality; limited generalizability
Orvieto et al.	Observational	7	6	High risk	Small sample size; high bias potential
Kahyaoglu et al.	Retrospective cohort	28	6	N/A	Moderate quality; retrospective limitations
Madendag et al.	Observational before-and-after	132	7	N/A	Good quality; robust before-and-after data
Gullo et al.	Prospective cohort	200	8	N/A	High quality; multicenter design

**Table 2:** Summary of the research articles used in this systematic review.

No	Title	Author	Sample Size	Characteristics	Correlation	Summary
1	SARS-CoV-2 infection negatively affects ovarian function in ART patients	Herrero et al.	80 women Control group n = 34, who had never experienced COVID and n = 46 post COVID-19 patients 2–9 months after infection	Age: 21–41 years old Undergoing ART BMI 23.43	Measure of MII oocytes, basal estradiol and progesterone, AMH, AFC, number of retrieved oocytes	For the first time it was shown that IgG antibodies against SARS-CoV-2 are present in follicular fluid. This fact changes the immunological environment in the ovary and can affect the maturation of oocytes
2	Past COVID-19: the impact on IVF outcomes based on follicular fluid lipid profile	Lomova et al.	237 women	68 with successful IVF 12 miscarried 103 without infection	Identifying the lipid profile in the follicular fluid of the women	A change in the lipid profile of the follicular fluid of the infected with COVID-19n eomrn was observed.
3	Ovulatory upregulation of angiotensin-converting enzyme 2, a receptor for SARS-CoV-2, in dominant follicles of the human ovary	Choi et al.	46 women	30 premenopausal women undergoing surgery for tubal ligation and 16 premenopausal women undergoing in vitro fertilization.	ACE2 mRNA in granulosa cells and cumulus cells	The data suggest the possible impact of COVID-19 on a vital cyclic event of ovarian function
4	Ovarian follicular function is not altered by SARS-CoV-2 infection or BNT162b2 mRNA COVID-19 vaccination	Bentov et al.	7 women	Aged 18–44 and exposed to COVID	AFC and anti-SARS-CoV-2 IgG in follicular fluid	a
5	Influence of COVID-19 infection/vaccination on menstrual regularity and hormonal function in Saudi females of reproductive age	Fouzeyyah Ali Alsaeed et al.	79 women who had been vaccinated and exposed to COVID-19	Aged 18–45 who had been vaccinated and exposed to COVID-19	Serum LH, FSH, TSH, estradiol, and prolactin levels; free T3 and T4; and vitamin D during the follicular phase of the menstrual cycle	It is noteworthy that irregular menstrual cycles were linked to higher levels of the prolactin hormone and lower levels of Vit. D.
6	Impact of omicron variant infection on female fertility and laboratory outcomes: a self-controlled study	Hu et al.	179 women	oocyte retrieval before and after SARS-CoV-2	FSH, LH, estrogen, AMH, AFC	After recovery, no significant change in IVF outcome
7	Does COVID-19 infection influence patients’ performance during IVF-ET cycle? An observational study	Orvieto et al.	7 women	Undergoing IVF	Oocytes per OPU and MII oocytes per OPU	Change in the number of matured oocytes
8	Does coronavirus disease-19 infection affect ovarian reserve in infertile women? A retrospective study	Kahyaoglu et al.	28 women	All infertile and aged 21–40	Serum FSH, LH, estradiol	In already infertile women, no significant changes were detected
9	COVID-19 disease does not cause ovarian injury in women of reproductive age: an observational before-and-after COVID-19 study	Madendag et al.	132 women	Aged 18–40	Serum FSH, LH, FSH/LH ratio, estradiol	Menstrual changes probably linked to inflammation and immune response during infection by SARS-CoV-2
10	COVID-19 and female fertility: an observational prospective multicenter cohort study: upholding reproductive rights in emergency circumstances	Gullo et al.	200 women	Aged 24–43 with diagnosis of infertility	Plasma levels of FSH, LH, estradiol, AMH, and antral follicular count	The researchers suggest that infection by SARS-CoV-2 may negatively affect reproductive health and may cause a low antral follicle count and reduced AMH
11	Analysis of sex hormones and menstruation in COVID-19 women of child-bearing age	Li et al.	237 women of childbearing age	Diagnosed with COVID-19	Blood samples from the early follicular phase were tested for sex hormones and anti-Müllerian hormone (AMH)	The researchers found that menstruation changes of the studied patients might be the consequence of transient sex hormone changes caused by suppression of ovarian function that quickly resume after recovery

## Data Availability

Data are contained within the article.
